# Co-mutation landscape and its prognostic impact on newly diagnosed adult patients with *NPM1*-mutated de novo acute myeloid leukemia

**DOI:** 10.1038/s41408-024-01103-w

**Published:** 2024-07-22

**Authors:** Yiyi Yao, Yile Zhou, Nanfang Zhuo, Wanzhuo Xie, Haitao Meng, Yinjun Lou, Liping Mao, Hongyan Tong, Jiejing Qian, Min Yang, Wenjuan Yu, De Zhou, Jie Jin, Huafeng Wang

**Affiliations:** 1 Department of Hematology, The First Affiliated Hospital, Zhejiang University School of Medicine, Hangzhou, 310003 Zhejiang PR China; 2 Zhejiang Provincial Key Laboratory of Hematopoietic Malignancy, Zhejiang University, Hangzhou, 310000 Zhejiang PR China; 3 Zhejiang Provincial Clinical Research Center for Hematological disorders, Hangzhou, 310000 Zhejiang PR China; 4 Zhejiang University Cancer Center, Hangzhou, 310000 Zhejiang PR China

**Keywords:** Cancer genomics, Cancer genomics, Genetics research

Dear Editor,

Approximately 25–35% of adult patients with acute myeloid leukemia (AML) carries *NPM1* mutation, which generally indicated a favorable outcome in the absence of *FLT3-ITD* mutation [1]. *NPM1* mutations are absent in clonal hematopoiesis, and have been considered as AML initiating lesions [2]. Research on co-mutation characteristics of *NPM1*-mutated patients concentrated on *FLT3-ITD*, which has been suggested to hold a negative prognostic impact on *NPM1*-mutated patients by several large retrospective clinical studies [3, 4]. Besides *FLT3-ITD*, although there remains controversy, other high-frequency co-mutations such as *DNMT3A*, *IDH1*, *IDH2*, *FLT3-TKD*, *NRAS*, and *WT1* mutations have also been pointed out to affect the prognosis of *NPM1-*mutated patients [3, 5–9]. Indeed, identification of specific co-mutation combinations other than *FLT3-ITD* mutation is essential for precise risk stratification and treatment strategy optimization for *NPM1*-mutated AML patients. Since allogeneic hematopoietic stem cell transplantation (allo-HSCT) is generally considered to improve the long-term outcome of most adverse-risk and suitable intermediate-risk AML patients, for *NPM1*-mutated AML patients, it is imperative to revisit the co-mutation profiles to determine the optimal population who may benefit from allo-HSCT.

In this study, we conducted a retrospective analysis of newly diagnosed adult AML patients with *NPM1* mutations (acute promyelocytic leukemia excluded) in our center diagnosed from October 2018 to December 2022, focusing on exploring the therapeutic and prognostic significance of co-mutation characteristics in AML patients with *NPM1* mutations. Patients who received at least one complete course of induction therapy were included in the further outcome analysis. Table S1 provided details of induction chemotherapy. We evaluated efficacy after two induction cycles, unless patients achieved CR/CRi after receiving only one induction cycle or discontinued treatment. Response evaluation was performed according to the NCCN guidelines for AML (version 3. 2023) and was categorized as CR/CRi or non-CR/CRi (including PR and NR) cohort [10]. Overall survival (OS) was defined as the time interval from treatment initiation until death due to any reason. Event-free survival (EFS) was defined as the time interval from treatment initiation to the occurrence of induction failure, relapse, or death, whichever came first. Disease-free survival (DFS) was defined as the time interval from disease remission to the occurrence of relapse or death, whichever came first. The study was conducted in accordance to the Declaration of Helsinki and was approved by the Ethics Committee of the First Affiliated Hospital of Zhejiang University College of Medicine (Hangzhou, China, Ethics Approval Number: IIT20240304A). All statistical analyses were performed using GraphPad Prism 7.0 software (GraphPad Software, CA, USA) and SPSS 23.0 (SPSS Inc., Chicago, IL).

One hundred ninety-two newly diagnosed *NPM1*-mutated AML patients detected through next-generation sequencing (NGS) were analyzed (Tables S2–S4). Twenty *NPM1* mutants were identified, most of which were located in exon 12 and manifested as 4 base pair duplication/insertion alteration. Seven non-exon 12 mutants were located in exon 5, 8, 9 and exon 11, respectively (Fig. 1A and Table S5). A total of 56 co-mutated genes were detected in the cohort (Fig. 1B). Co-mutated genes with a detection rate of ≥10% included *FLT3* (56.77%), *DNMT3A* (48.44%), *TET2* (29.69%), *IDH2* (23.96%), *IDH1* (14.58%), *PTPN11* (11.46%), and *NRAS* (11.46%). Co-mutated genes related to epigenetics and signal transduction were the most common by functional classification (Table S6).

**Fig. 1:**
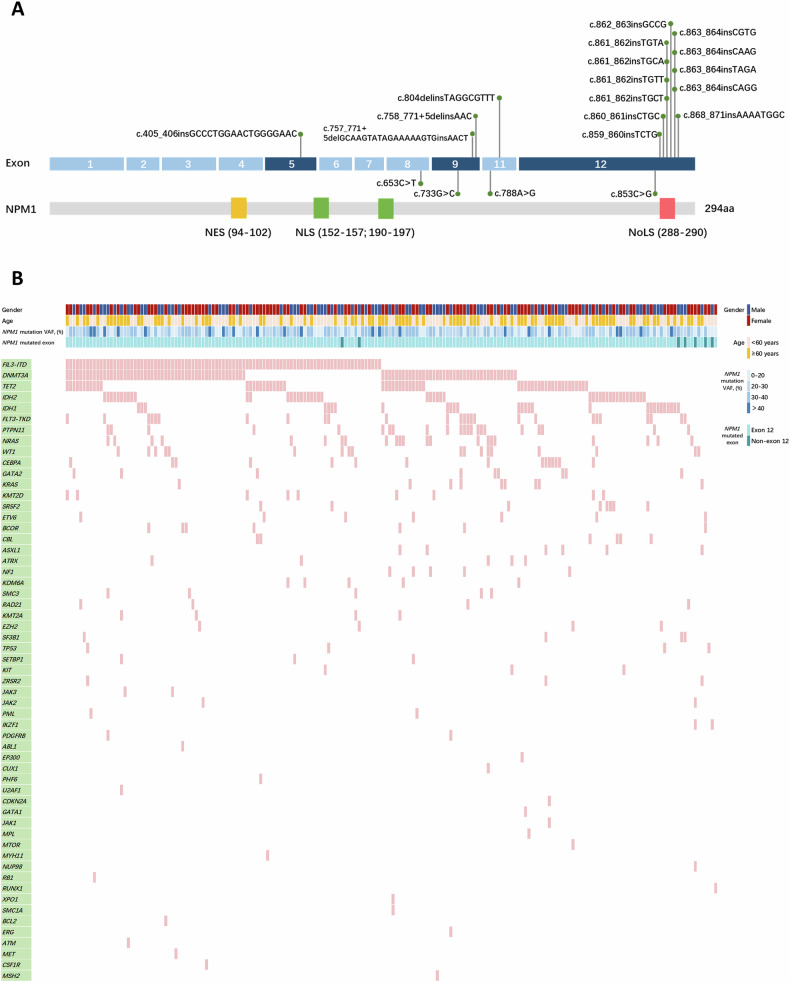
Landscape of *NPM1* mutations in adult AML. **A** Protein domain structure and location of amino acids affected by mutations in *NPM1*. Several nuclear import and export signals of NPM1 assist its nucleocytoplasmic shuttling and cytological localization. The conserved N-terminal domain of NPM1 contains a leucine-rich nuclear export signal (NES). The middle domain contains two nuclear localization signals (NLS) that drive NPM1 to move from the cytoplasm to the nucleus. The C-terminus contains a nucleolar localization signal (NoLS), in which two highly conserved tryptophan residues (W288 and W290) are responsible for the correct folding of the helix to stabilize the hydrophobic core of NoLS. Most of the insertion mutations in exon 12 led to the loss of the original NoLS signal and generated a new NES signal, leading to aberrant cytoplasmic dislocation of NPM1 protein. **B** Co-mutation distribution map of *NPM1*-mutated AML patients.

One hundred seventy-eight patients (92.71%) received at least one complete course of intensive induction chemotherapy and underwent efficacy assessment, of which 133 patients (74.72%) achieved CR/CRi within two courses of induction chemotherapy. The median follow-up of the 178 patients was 26.23 months (95% confidence interval [CI], 23.31–29.16). The median OS and DFS have not been reached, with the median EFS of 15.03 months (95% CI, 8.25–21.82). The 3-year expected OS, EFS, and DFS were 51.5%, 40.3%, and 53.7%, respectively.

Regardless of the cut-off value of variant allele frequency (VAF) levels, there was no significant difference in OS, EFS, and DFS between *NPM1*^low VAF^ group and *NPM1*^high VAF^ group (Fig. S1). Then we focused on impact of co-mutations on response and outcome of AML patients with *NPM1* mutations. Among the 178 *NPM1*-mutated patients included in the follow-up, we noticed that patients with either *FLT3-ITD* or *DNMT3A* mutations showed significantly worse CR/CRi rates and prognosis trends than wild type group (*FLT3-ITD*, CR/CRi rates, 63.41% vs. 84.38%, *p* = 0.001; median OS, 14.3 months vs. NR, *p* < 0.001; median EFS, 7.3 months vs. NR, *p* < 0.001; median DFS, 21.6 months vs. NR, *p* = 0.044; *DNMT3A*, CR/CRi rates, 67.44% vs. 81.53%, *p* = 0.013; median OS, 15.3 months vs. NR, *p* < 0.001; Median EFS, 11.6 months vs 27.7 months, *p* = 0.031; Median DFS, *p* = 0.337) (Table S7 and Fig. S2). We further divided patients into four subgroups according to the *FLT3-ITD* and *DNMT3A* mutation status. *NPM1/FLT3-ITD/DNMT3A* triple mutants showed extremely poor OS and EFS trends among four groups (Fig. 2A, B). Besides, we noticed that when combined with *DNMT3A* mutations, *FLT3-ITD* mutated patients exhibited significantly worse OS than that of *FLT3-ITD* wild-type patients (*p* = 0.003), while similar results were found in *DNMT3A* wild-type patients (*p* = 0.002); We also noticed that when combined with *FLT3-ITD* mutations, *DNMT3A* mutated patients exhibited significantly worse OS than that of *DNMT3A* wild-type patients (*p* = 0.045), with similar results occurred in *FLT3-ITD* wild-type patients (*p* = 0.020) (Fig. 2A).

**Fig. 2:**
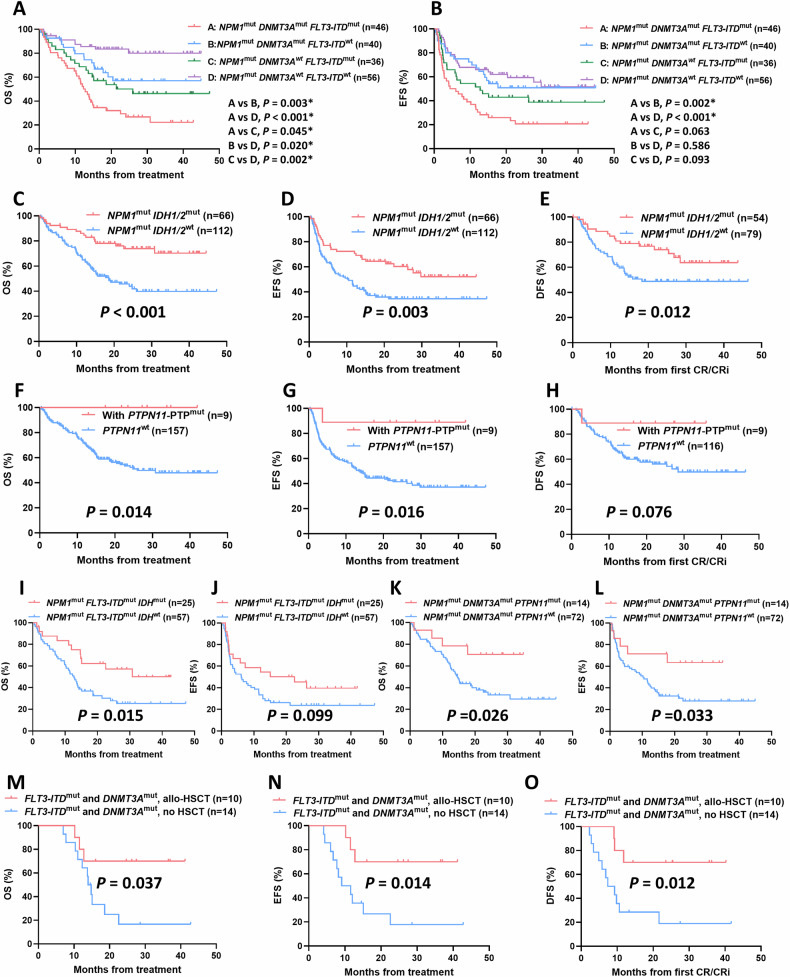
Outcome of *NPM1*-mutated AML patients with diverse co-mutation profiles. **A** OS and **B** EFS of *NPM1*-mutated AML patients with different combination patterns of *FLT3-ITD* and *DNMT3A* mutations. **C** OS, **D** EFS, and **E** DFS of *NPM1*-mutated AML patients with *IDH1/2* mutation**. F** OS, **G** EFS, and **H** DFS of *NPM1*-mutated AML patients with *PTPN11*-PTP mutation. **I** OS and **J** EFS of *NPM1*^mut^*FLT3-ITD*^mut^ AML patients with *IDH* mutations. **K** OS and **L** EFS of *NPM1*^mut^*DNMT3A*^mut^ AML patients with *PTPN11* mutations. **M** OS, **N** EFS, and **O** DFS of allo-HSCT on *NPM1*-mutated AML patients harbored both *FLT3-ITD* and *DNMT3A* mutations.

For patients combined with *IDH1/2* mutations, we observed that the *IDH1/2* mutant group significantly improved OS, EFS, and DFS compared with wild-type group (Median OS, NR vs. 18.6 months, *p* < 0.001; Median EFS, NR vs 10.2 months, *p* = 0.003; Median DFS, NR vs 18.3 months, *p* = 0.012) (Figs. 2C–E and S3). Although patients combined with *PTPN11* mutations showed a trend toward improved outcome compared with *PTPN11* wild-type, the difference was not significant (Fig. S4). *PTPN11* mutations have been reported to be mainly clustered in the N-terminal Src homology region 2 (N-SH2) and phosphatase (PTP) domains. Since mutations in both two domains involved in attenuating the autoinhibition of the protein, SHP2, encoded by *PTPN11* [11], we further investigated whether mutations in different domains of *PTPN11* led to comparable outcome. The OS and EFS of patients with *PTPN11*-PTP domain mutations were significantly improved compared to those with *PTPN11* wild-type (Median OS, NR vs 26.0 months, *p* = 0.014; Median EFS, NR vs 13.5 months, *p* = 0.016). Similar trends were found in DFS, whereas patients with *PTPN11*-N-SH2 domain mutations showed no significant improvement in outcome (Figs. 2F–H and S4). In addition, Fig. S5 showed the prognostic impact of other co-mutation genes with a detection rate of ≥10% in the follow-up patients, including *TET2*, *FLT3-TKD*, *NRAS*, and *WT1*, with trends all non-significant.

Further, we took into account the presence of *IDH* or *PTPN11* mutations in *NPM1-*mutated patients combined with *FLT3-ITD* or *DNMT3A* mutations to explore the prognostic impact of the specific co-mutation interaction patterns. Separately, carrying *IDH* mutations significantly improved OS and exhibited an improved EFS trend in patients with *NPM1*/*FLT3-ITD* dual mutations (Median OS, 30.8 vs 12.8 months, *p* = 0.015; Median EFS, 22.6 vs 6.1 months, *p* = 0.099), but has no significant impact on the outcome of patients with *NPM1*/*DNMT3A* mutations (Figs. 2I, J and S6). Similarly, carrying *PTPN11* mutations significantly improved OS and EFS in patients with *NPM1*/*DNMT3A* dual mutations (Median OS, NR vs. 14.6 months, *p* = 0.026; Median EFS, NR vs. 10.2 months, *p* = 0.033), but has no significant impact on the outcome of patients with *NPM1*/*FLT3-ITD* mutations (Figs. 2K, L and S6).

Previous research generally acknowledged that allo-HSCT is beneficial for *FLT3-ITD* mutated AML patients without *NPM1* mutations. To identify the subgroup of *NPM1*-mutated AML patients likely to benefit from allo-HSCT, we explored the prognosis of patients who underwent allo-HSCT during post-remission after achieving CR/CRi within two courses of induction. A total of 32 patients received allo-HSCT, with another four patients relapsed and received salvage-HSCT during post-remission. For patients with *NPM1* mutation, receiving allo-HSCT or salvage-HSCT did not significantly improve the outcome compared with non-transplanted patients (Fig. S7). For patients with *NPM1* mutations combined with either *FLT3-ITD* or *DNMT3A* mutation, allo-HSCT showed a trend toward improved outcome, but the difference was not significant. When further focused on patients with *NPM1/FLT3-ITD/DNMT3A* triple mutations characterized by poor prognosis, we observed that allo-HSCT significantly improved the OS, EFS, and DFS of these subgroup (Median OS, NR vs. 14.0 months, *p* = 0.037; Median EFS, NR vs. 9.1 months, *p* = 0.014; Median DFS, NR vs. 7.4 months, *p* = 0.012) (Fig. 2M–O). Nevertheless, for *NPM1*-mutated patients with wild type *FLT3-ITD* and *DNMT3A*, administration of allo-HSCT showed no improved outcome (Fig. S7).

Our results indicated that in *NPM1*-mutated AML, co-mutations of *IDH1*/2 and *PTPN11*-PTP domain were correlated with favorable prognosis, whereas *FLT3-ITD* and *DNMT3A* co-mutations were indicative of poor prognosis. Notably, the presence of *NPM1/FLT3-ITD/DNMT3A* triple mutations is associated with exceptionally adverse OS and EFS trends. Several studies have reported *NPM1*/*FLT3-ITD*/*DNMT3A*, the most common triple mutation pattern in *NPM1*-mutated patients, defined an AML subgroup with extremely poor prognosis [7, 12], which aligned with our findings. Further, our results on specific co-mutation combinations indicated that *IDH* and *PTPN11* co-mutations, respectively, ameliorated the adverse prognosis of patients with *NPM1*/*FLT3-ITD* or *NPM1*/*DNMT3A* dual mutations, thus two subsets with improved prognosis were redefined from the original adverse-prognosis subset of *NPM1*-mutated AML. Besides, for patients with *NPM1*/*FLT3-ITD* dual mutations, allo-HSCT post-first remission has demonstrated a significant enhancement in both OS and DFS juxtaposed with the continued administration of chemotherapy alone [13, 14]. However, another large cohort study on pediatric AML reported opposite results [15]. Our research endeavored to identify the optimal population who may benefit from allo-HSCT. The findings underscored the therapeutic potential of allo-HSCT, particularly for AML patients with *NPM1/FLT3-ITD/DNMT3A* triple mutations during post-remission.

In summary, these findings underscored the importance of co-mutation analysis in *NPM1*-mutated AML for risk stratification and therapeutic decision-making, suggesting that allo-HSCT may be a recommended strategy for *NPM1*-mutated patients with specific adverse co-mutation profiles. Nevertheless, further research is needed to confirm these findings and explore how these co-mutations interact to diversify the outcome of *NPM1*-mutated AML patients.

### Supplementary information

Supplementary materials

## Data Availability

The data are not publicly available, owing to ethics considerations and privacy restriction, but can be requested from the corresponding author if necessary.
