# Supplementary material for: Co-mutation landscape and its prognostic impact on newly diagnosed adult patients with NPM1-mutated de novo acute myeloid leukemia
Source: Blood Cancer J. 2024 Jul 22;14(1):118. doi: 10.1038/s41408-024-01103-w (PMC11263537; doi:10.1038/s41408-024-01103-w)
Supplement: Supplementary file 1 — Supplementary materials [file 41408_2024_1103_MOESM1_ESM.docx]

**Supplemental Information to: Co-mutation landscape and its prognostic impact on newly diagnosed adult patients with *NPM1*-mutated de novo acute myeloid leukemia**

**Table S1. Induction regimen of cycle 1 and cycle 2.**

| **Induction regimen classification** | **Cycle 1, n (%)** | **Cycle 2, n (%)** |
| --- | --- | --- |
| Standard-intensity chemotherapy | 67 (37.64%) | 17 (26.56%) |
| Reduced-intensity chemotherapy/ Targeted therapy | 16 (8.99%) | 7 (10.94%) |
| Venetoclax plus standard-intensity chemotherapy | 40 (22.47%) | 5 (7.81%) |
| Venetoclax plus reduced-intensity chemotherapy/ Targeted therapy | 55 (30.90%) | 35 (54.69%) |
| Total | 178 | 64 |

**Table S2. Baseline characteristics.**

| **Characteristics** |  | **AML patients with *NPM1* mutations (n=192)** |
| --- | --- | --- |
| **Age, years** | Median (IQR) | 59 (51-68) |
| **Gender** | M, n (%) | 86 (44.79) |
|  | F, n (%) | 106 (55.21) |
| **FAB diagnosis** | M0, n (%) | 2 (1.04) |
|  | M1, n (%) | 25 (13.02) |
|  | M2, n (%) | 80 (41.67) |
|  | M4, n (%) | 6 (3.13) |
|  | M5, n (%) | 79 (41.15) |
| **WBC count, × 10^9^/L** | Median (IQR) | 32.10 (5.58-74.06) |
| **HB, g/L** | Median (IQR) | 83.50 (68.00-99.75) |
| **PLT, × 10^9^/L** | Median (IQR) | 70.00 (44.50-106.00) |
| **Baseline BM blasts** | Median (IQR) | 73.00% (58.00%-84.00%) |
| **Karyotype** | NK, n (%) | 141 (73.44) |
|  | AK, n (%) | 23 (11.98) |
|  | No mitotic figures, n (%) | 14 (7.29) |
|  | Unknown, n (%) | 14 (7.29) |
| **Cytogenetic risk category** | Favorable, n (%) | 128 (66.67) |
|  | Intermediate, n (%) | 15 (7.81) |
|  | Adverse, n (%) | 2 (1.04) |
|  | Unknown, n (%) | 47 (24.48) |
| **ELN2022 risk category** | Favorable, n (%) | 61 (31.77) |
|  | Intermediate, n (%) | 71 (36.98) |
|  | Adverse, n (%) | 13 (6.77) |
|  | Unknown, n (%) | 47 (24.48) |
| **VAF of *NPM1* mutation** | Median (IQR) | 29.70% (19.80%-36.63%) |

Abbreviations: M, male; F, female; FAB, French-American-British classification system; WBC, white blood cell; HB, hemoglobin; PLT, platelet; BM, bone marrow; NK, normal karyotype, AK, abnormal karyotype; ELN, European Leukemia Net; VAF, variant allele frequency.

**Table S3. Next generation sequencing gene panel.**

| ***ABL1*** | ***BCOR*** | ***CUX1*** | ***FIP1L1*** | ***IKZF1*** | ***MET*** | ***NSD1*** | ***PTEN*** | ***SMC3*** | ***TP53*** | ***RAD21*** |
| --- | --- | --- | --- | --- | --- | --- | --- | --- | --- | --- |
| ***AKT1*** | ***BCR*** | ***DNMT3A*** | ***FLT3*** | ***JAK1*** | ***MPL*** | ***NUP98*** | ***PTPN11*** | ***SRSF2*** | ***U2AF1*** | ***SMC1A*** |
| ***AKT2*** | ***CALR*** | ***EP300*** | ***GATA1*** | ***JAK2*** | ***MSH2*** | ***PDGFRA*** | ***RARA*** | ***STAG2*** | ***WT1*** | ***KDM2B*** |
| ***AKT3*** | ***CBL*** | ***ERG*** | ***GATA2*** | ***JAK3*** | ***MTOR*** | ***PDGFRB*** | ***RB1*** | ***STAT3*** | ***XPO1*** | ***KDM5A*** |
| ***APC*** | ***CDKN2A*** | ***ETV6*** | ***GATA3*** | ***KIT*** | ***MYC*** | ***PHF6*** | ***RUNX1*** | ***STAT5A*** | ***ZRSR2*** | ***KDM6A*** |
| ***ASXL1*** | ***CEBPA*** | ***EWSR1*** | ***HRAS*** | ***KMT2A*** | ***NF1*** | ***PIK3CA*** | ***MTG8*** | ***STAT5B*** | ***PML*** | ***SRC*** |
| ***ATM*** | ***CSF1R*** | ***EZH2*** | ***IDH1*** | ***KMT2D*** | ***NPM1*** | ***PRDM1*** | ***SETBP1*** | ***STAT6*** | ***CBFB*** | ***BCL2*** |
| ***ATRX*** | ***CSF3R*** | ***FGFR1*** | ***IDH2*** | ***KRAS*** | ***NRAS*** | ***PTCH1*** | ***SF3B1*** | ***TET2*** | ***MYH11*** | ***SMO*** |

**Table S4. Panel of gene fusions.**

| ***BCR*::*ABL*** | ***KMT2A*::*AF4*** | ***ETV6*::*ABL*** | ***NPM*::*RARa*** | ***KMT2A*::*ENL*** | ***NUP98*::*HoxA11*** |
| --- | --- | --- | --- | --- | --- |
| ***CBFB*::*MYH11*** | ***KMT2A*::*AF6*** | ***ETV6*::*RUNX1*** | ***NUMA1*::*RARa*** | ***KMT2A*::*ELL*** | ***NUP98*::*HoxA13*** |
| ***PML*::*RARa*** | ***KMT2A*::*AF9*** | ***ETV6*::*JAK2*** | ***RUNX1*::*MTG8*** | ***KMT2A*::*AFX*** | ***NUP98*::*HoxC11*** |
| ***FIP1L1*::*RARa*** | ***KMT2A*::*AF10*** | ***ETV6*::*PDGFRB*** | ***RUNX1*::*MDS/EVI1*** | ***KMT2A*::*SEPT6*** | ***NIP98*::*HoxD13*** |
| ***PLZF*::*RARa*** | ***KMT2A*::*AF17*** | ***FIP1L1*::*PDGFRA*** | ***RUNX1*::*MTG16*** | ***SIL*::*TAL1*** | ***NUP98*::*PMX1*** |
| ***PRKAR1A*::*RARa*** | ***KMT2A*::*AF1p*** | ***ETV6*::*PDGFRa*** | ***NPM*::*MLF1*** | ***E2A*::*PBX1*** | ***TLS*::*ERG*** |
| ***STAT5b*::*RARa*** | ***KMT2A*::*AF1q*** | ***NUP98*::*HoxA9*** | ***DEK*::*NUP214*** | ***E2A*::*HLF*** | ***SET*:: *NUP214*** |

**Table S5. *NPM1* mutants in 192 AML patients.**

| **Mutation type** | **Exon** | **Frequency, n (%)** |
| --- | --- | --- |
| c.859_860insTCTG (Type A) | 12 | 147 (76.56%) |
| c.860_861insCTGC (Type D) | 12 | 15 (7.81%) |
| c.861_862insTGTT | 12 | 7 (3.65%) |
| c.861_862insTGCT | 12 | 4 (2.08%) |
| c.861_862insTGCA (Type B) | 12 | 3 (1.56%) |
| c.863_864insCAGG | 12 | 2 (1.04%) |
| c.861_862insTGTA | 12 | 1 (0.52%) |
| c.863_864insTAGA | 12 | 1 (0.52%) |
| c.863_864insCAAG | 12 | 1 (0.52%) |
| c.862_863insGCCG | 12 | 1 (0.52%) |
| c.863_864insCGTG | 12 | 1 (0.52%) |
| c.405_406insGCCCTGGAACTGGGGAAC | 5 | 1 (0.52%) |
| c.653C>T | 8 | 1 (0.52%) |
| c.733G>C | 9 | 1 (0.52%) |
| c.757_771+5delGCAAGTATAGAAAAAGTGinsAACT | 9 | 1 (0.52%) |
| c.758_771+5delinsAAC | 9 | 1 (0.52%) |
| c.788A>G | 11 | 1 (0.52%) |
| c.804delinsTAGGCGTTT | 11 | 1 (0.52%) |
| c.859_860insTCTG；c.853C>G | 12 | 1 (0.52%) |
| c.868_871insAAAATGGC | 12 | 1 (0.52%) |

**Table S6. Co-mutated gene function.**

| **Gene function** | | **Mutated gene (n)** |
| --- | --- | --- |
| **DNA damage response** |  | *MSH2* (1), *TP53* (2) |
| **Epigenetics** | **DNA methylation** | *DNMT3A* (93), *IDH1* (28), *IDH2* (46), *TET2* (57) |
|  | **Chromatin remodeling** | *ATRX* (5) |
|  | **Histone methylation** | *ASXL1* (6), *EZH2* (4), *KDM6A* (5), *KMT2A* (4), *KMT2D* (9) |
|  | **Histone acetylation** | *EP300* (1) |
|  | **Other epigenetic-related genes** | *BCOR* (7), *PHF6* (1), *SETBP1* (3) |
| **Signal transduction** | **JAK-STAT signaling** | *JAK1* (1), *JAK2* (2), *JAK3* (2) |
|  | **RAS-MAPK signaling** | *CBL* (6), *KRAS* (9), *NF1* (5), *NRAS* (22), *PTPN11* (22) |
|  | **mTOR signaling** | *MTOR* (1) |
|  | **Cytokine receptor** | *CSF1R* (1), *FLT3* (109), *KIT* (3), *MET* (1), *MPL* (1), *PDGFRB* (2) |
| **Transcription factor** |  | *CEBPA* (15), *CUX1* (1), *ETV6* (7), *ERG* (1), *GATA1* (1), *GATA2* (9), *IKZF1* (2), *RUNX1* (1), *WT1* (18) |
| **Splicing factors** |  | *SF3B1* (4), *SRSF2* (8), *U2AF1* (1), *ZRSR2* (3) |
| **Cohesin complex gene** |  | *RAD21* (4), *SMC3* (5), *SMC1A* (1) |
| **Cell cycle regulation** |  | *ATM* (1), *CDKN2A* (1) |
| **Others** |  | *ABL1* (1), *BCL2* (1), *PML* (2), *MYH11* (1), *NUP98* (1), *RB1* (1), *XPO1* (1) |

**Table S7. Response assessment.**

| **Characteristics on mutations** |  | **CR/CRi, n (%)** | ***P* value** |
| --- | --- | --- | --- |
| **Total, n = 178** |  | 133 (74.72%) |  |
| **VAF of *NPM1* mutation** | Low (<30%, n = 89) | 73 (82.02%) | 0.025 |
|  | High (≥30%, n = 89) | 60 (67.41%) |  |
| ***FLT3-ITD*** | *FLT3-ITD*^mut^ (n = 82) | 52 (63.41%) | 0.001 |
|  | *FLT3-ITD*^wt^ (n = 96) | 81 (84.38%) |  |
| ***DNMT3A*** | *DNMT3A* ^mut^ (n = 86) | 58 (67.44%) | 0.013 |
|  | *DNMT3A* ^wt^ (n = 92) | 75 (81.52%) |  |
| ***IDH1*** | *IDH1*^mut^ (n = 26) | 21 (80.77%) | 0.442 |
|  | *IDH1*^wt^ (n = 152) | 112 (73.68%) |  |
| ***IDH2*** | *IDH2*^mut^ (n = 41) | 34 (82.93%) | 0.168 |
|  | *IDH2*^wt^ (n = 137) | 99 (72.26%) |  |
| ***PTPN11*** | *PTPN11*^mut^ (n = 21) | 17 (80.95%) | 0.484 |
|  | *PTPN11*^wt^ (n = 157) | 116 (73.89%) |  |

Abbreviations: CR/CRi, complete remission/complete remission with incomplete blood cell count recovery; VAF, variant allele frequency.

**Figure legends**


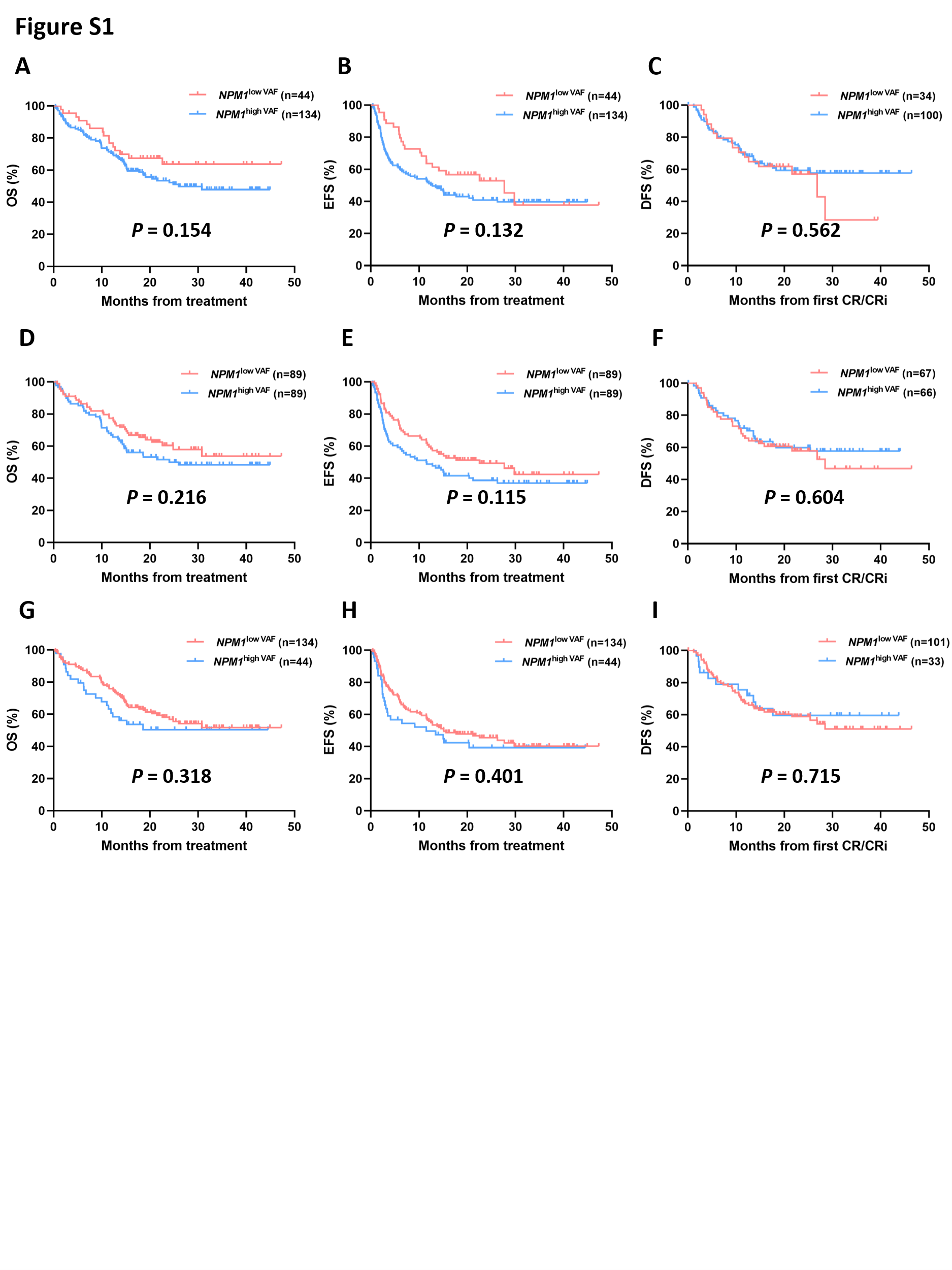


**Figure S1. Outcome of *NPM1*-mutated AML patients with different levels of *NPM1* mutation VAF.**

**(A)** OS, **(B)** EFS, and **(C)** DFS of *NPM1*-mutated AML patients with cut-off for VAF levels of 0.25. **(D)** OS, **(E)** EFS, and **(F)** DFS of *NPM1*-mutated AML patients with cut-off for VAF levels of 0.5. **(G)** OS, **(H)** EFS, and **(I)** DFS of *NPM1*-mutated AML patients with cut-off for VAF levels of 0.75.


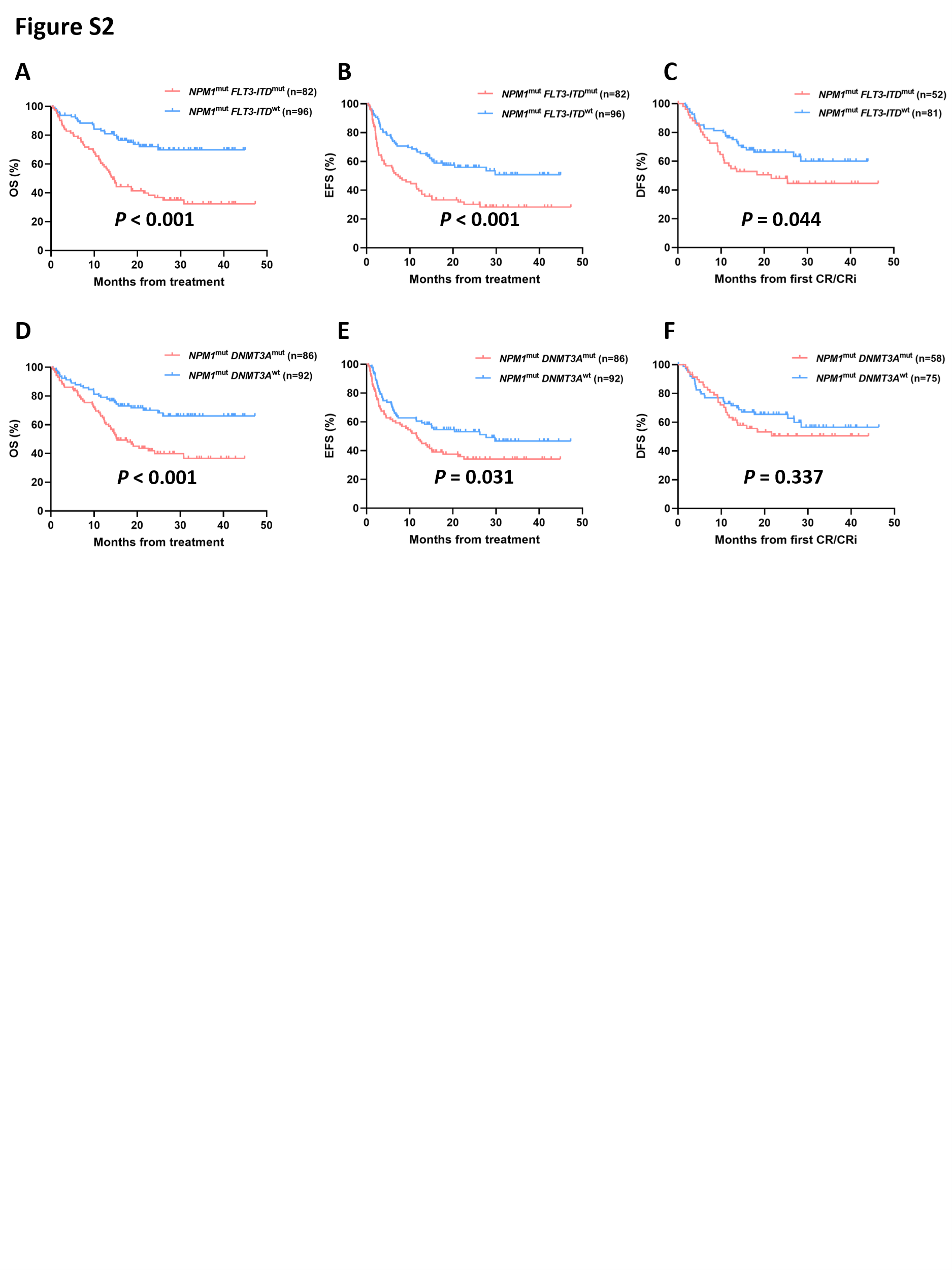


**Figure S2. Outcome of *NPM1*-mutated AML patients with *FLT3-ITD* or *DNMT3A* mutations.**

**(A)** OS, **(B)** EFS, and **(C)** DFS of *NPM1*-mutated AML patients with *FLT3-ITD*. **(D)** OS, **(E)** EFS, and **(F)** DFS of *NPM1*-mutated AML patients with *DNMT3A* mutation**.**


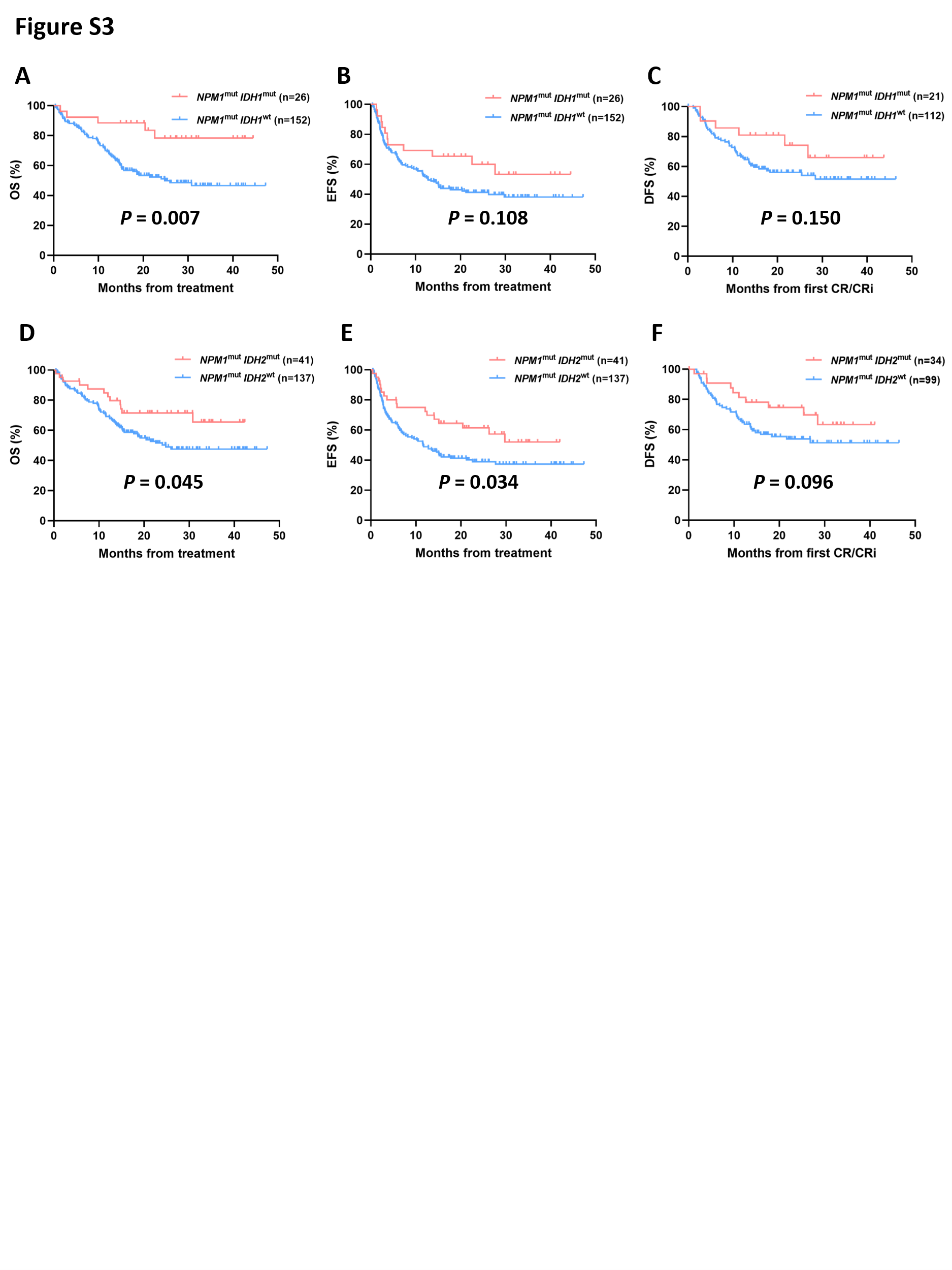


**Figure S3. Outcome of *NPM1*-mutated AML patients with *IDH1* or *IDH2* mutation.**

**(A**) OS, **(B)** EFS, and **(C)** DFS of *NPM1*-mutated AML patients with *IDH1* mutation. **(D)** OS, **(E)** EFS, and **(F)** DFS of *NPM1*-mutated AML patients with *IDH2* mutation**.**


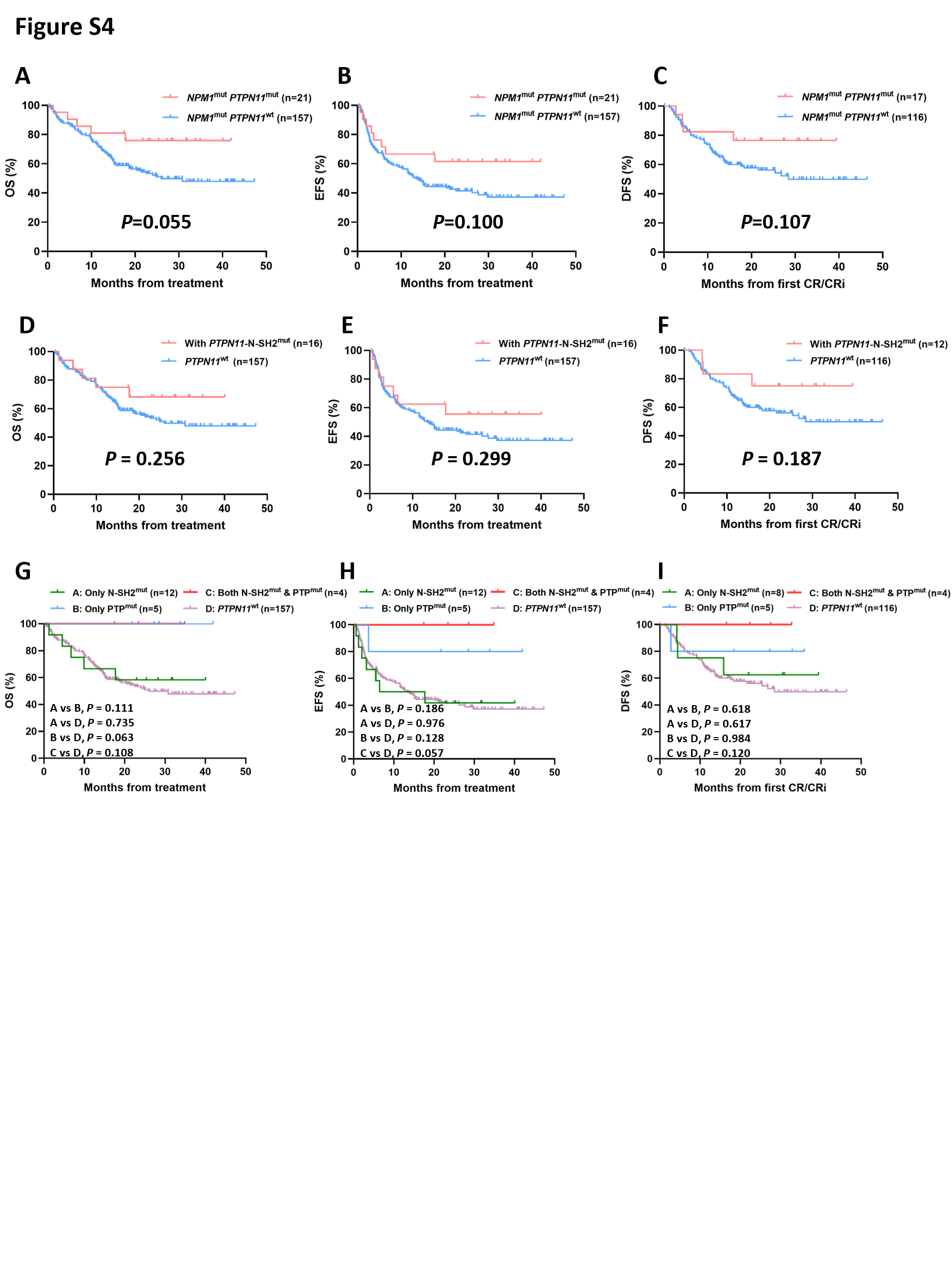


**Figure S4. Outcome of *NPM1*-mutated AML patients with *PTPN11* mutation.**

**(A)** OS, **(B)** EFS, and **(C)** DFS of *NPM1*-mutated AML patients with *PTPN11* mutation. **(D)** OS, **(E)** EFS, and **(F)** DFS of *NPM1*-mutated AML patients with *PTPN11*-N-SH2 mutation. **(G)** OS, **(H)** EFS, and **(I)** DFS of *NPM1*-mutated AML patients with different domain of *PTPN11* (N-SH2 or PTP domain) mutation.


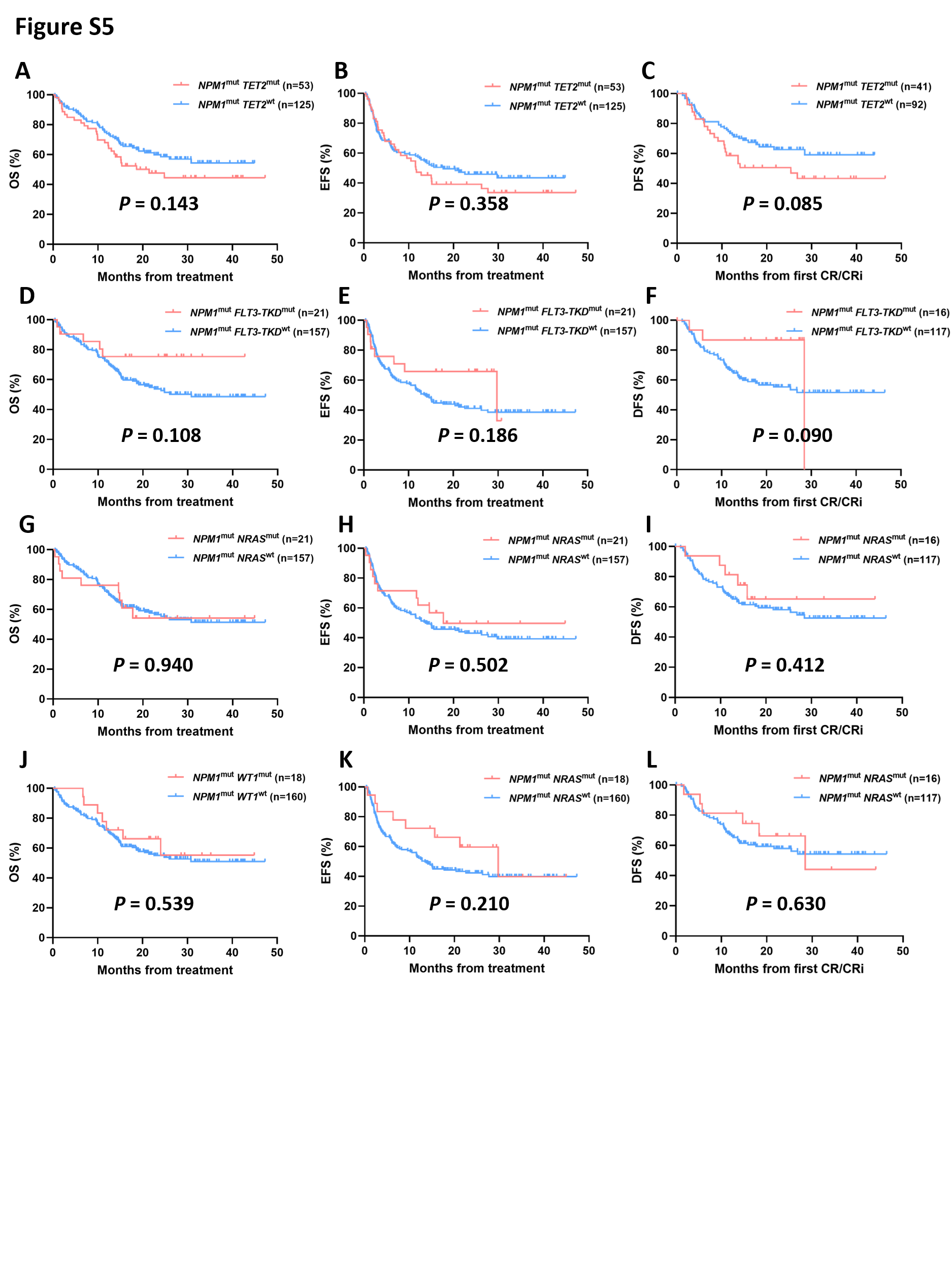


**Figure S5. Outcome of *NPM1*-mutated AML patients with other high-frequency mutations.**

**(A)** OS, **(B)** EFS, and **(C)** DFS of *NPM1*-mutated AML patients with *TET2* mutation. **(D)** OS, **(E)** EFS, and **(F)** DFS of *NPM1*-mutated AML patients with *FLT3-TKD* mutation**. (G)** OS, **(H)** EFS, and **(I)** DFS of *NPM1*-mutated AML patients with *NRAS* mutation**. (J)** OS, **(K)** EFS, and **(L)** DFS of *NPM1*-mutated AML patients with *WT1* mutation**.**


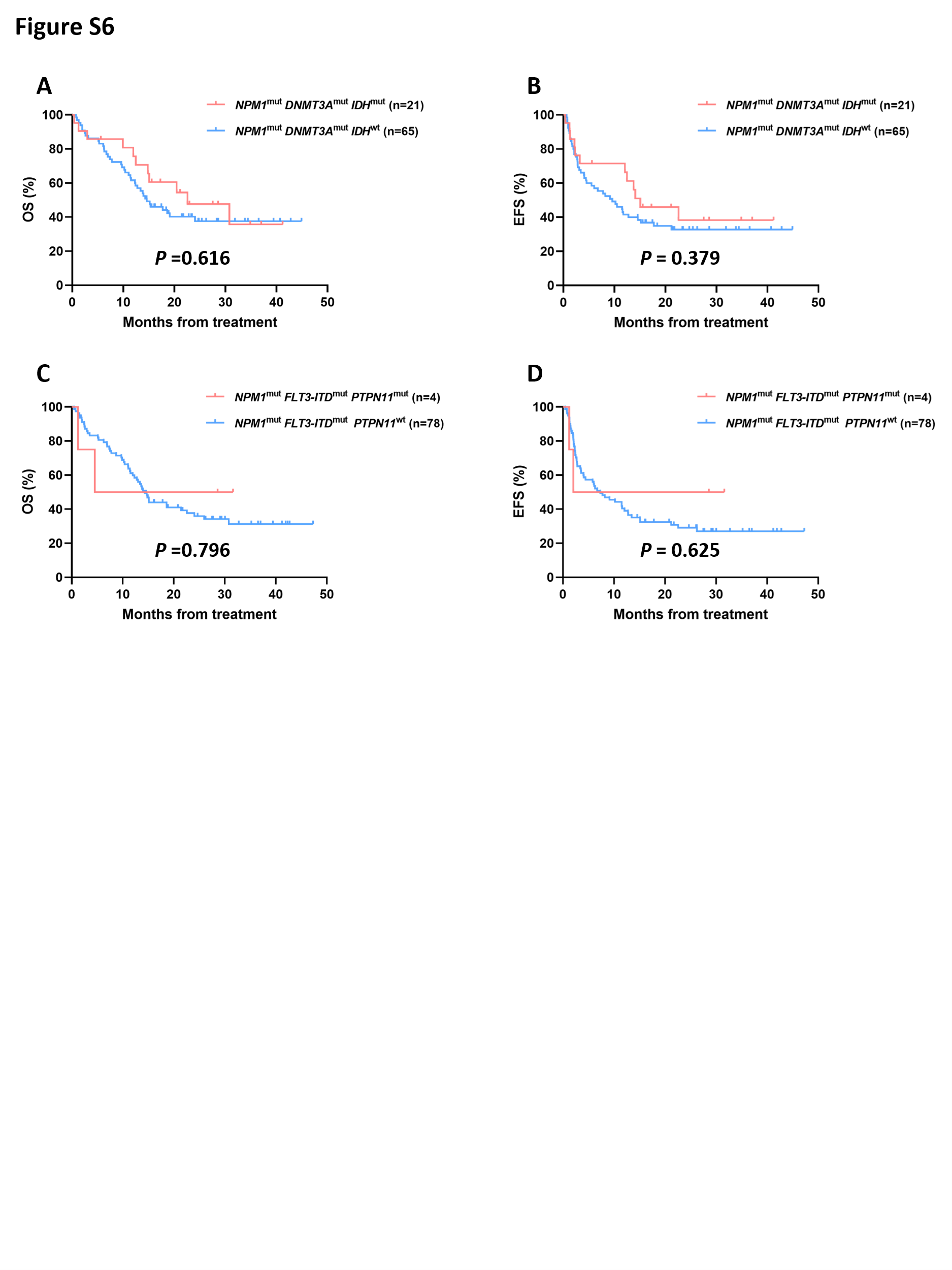


**Figure S6. Outcome of *NPM1*-mutated AML patients with different co-mutation combinations.**

**(A)** OS and **(B)** EFS of *NPM1*^mut^*DNMT3A*^mut^ AML patients with *IDH* mutations. **(C)** OS and **(D)** EFS of *NPM1*^mut^*FLT3-ITD*^mut^ AML patients with *PTPN11* mutations.


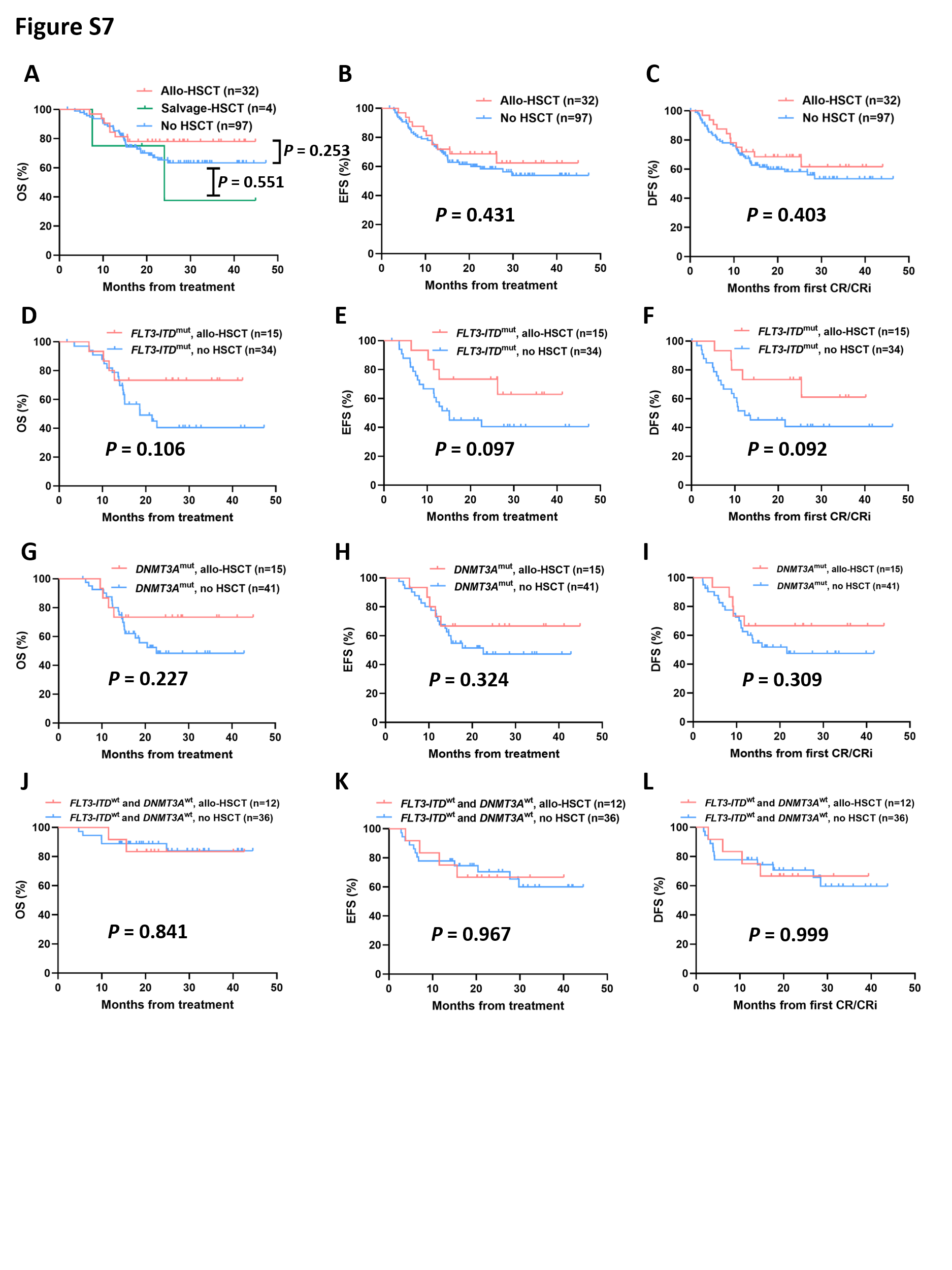


**Figure S7. Prognostic impact of allo-HSCT on *NPM1*-mutated AML patients who achieved CR/CRi within two courses of induction.**

**(A)** OS, **(B)** EFS, and **(C)** DFS of allo-HSCT on total *NPM1*-mutated AML patients. **(D)** OS, **(E)** EFS, and **(F)** DFS of allo-HSCT on *NPM1*-mutated AML patients combined with *FLT3-ITD* mutation. **(G)** OS, **(H)** EFS, and **(I)** DFS of allo-HSCT on *NPM1*-mutated AML patients combined with *DNMT3A* mutations. **(J)** OS, **(K)** EFS, and **(L)** DFS of allo-HSCT on *FLT3-ITD* and *DNMT3A* wild-type *NPM1*-mutated AML patients*.*
